# P38 MAPK/AKT signalling is involved in IL-33-mediated anti-apoptosis in childhood acute lymphoblastic leukaemia blast cells

**DOI:** 10.1080/07853890.2021.1970217

**Published:** 2021-08-26

**Authors:** Yiqian Wang, Hanyi Hou, Zhongping Liang, Xuexin Chen, Xindan Lian, Jie Yang, Zeyu Zhu, Huanmin Luo, Haibo Su, Qing Gong

**Affiliations:** a The Sixed Affiliated Hospital, GMU-GIBH Joint School of Life Sciences, Guangzhou Medical University, Guangzhou, China; b The Second Clinical Medicine School, Guangzhou Medical University, Guangzhou, China; c School of Pediatrics, Guangzhou Medical University, Guangzhou, China; d KingMed School of Laboratory Medicine, Guangzhou Medical University, Guangzhou, China; e The Third Clinical Medicine School, Guangzhou Medical University, Guangzhou, China

**Keywords:** ALL, IL1RL1, IL-33, P38 MAPK, AKT, anti-apoptosis

## Abstract

**Background:**

Acute lymphoblastic leukaemia (ALL) is often characterized by broad clinical and biological heterogeneity, as well as recurrent genetic aberrations. Despite remarkable improvements in the treatment outcome in paediatric ALL over the past several decades, it remains a leading cause of morbidity and mortality among children. Cytokines have been extensively studied in haematologic diseases; however, the mechanisms by which cytokines contribute to ALL pathogenesis remain poorly understood.

**Methods:**

IL-33 levels were measured by enzyme-linked immunosorbent assay (ELISA). IL1RL1 expression on ALL cell surface was accessed by flow cytometry. Expression of phosphorylated p38 MAPK, p38, pAKT, AKT and GAPDH were quantified by western blot. Cell survival signals were evaluated by apoptosis using flow cytometry.

**Results:**

BM samples from ALL patients at diagnosis upregulated their cell surface expression of IL1RL1, and a higher interleukin (IL)-33 level in the serum was observed as compared to the healthy individuals. Moreover, exogenous IL-33 treatment significantly inhibited apoptosis by activating p38 mitogen-activated protein kinase (MAPK) and AKT pathway, while the inhibitor for p38 MAPK, SB203580, counteracted IL-33-induced anti-apoptosis *via* inactivation of p38 MAPK and AKT. Furthermore, IL-33 negatively regulates cyclin B1 protein level while increasing the expression of CDK1, with SB203580 inhibiting the effect.

**Conclusion:**

Our study reveals an important role for IL-33/IL1RL1 axis in supporting ALL which may represent a novel treatment for paediatric patients.

**KEY MESSAGES**
- Both IL-33 and IL1RL1 levels are upregulated in primary ALL samples.
- IL-33 increased both p38 MAPK and AKT activation in ALL.
- IL-33 promotes survival and cell cycle progression of ALL cells via activating p38 MAPK.

## 1. Introduction

Acute lymphoblastic leukaemia (ALL), the most common malignancy in children and adolescents, is often characterized by a differentiation block and clonal proliferation of T or B lymphoblasts through the accumulation of immature lymphocytes [1,2]. ALL can occur in both children and adults, with a peak incidence between 1 and 4 years of age [3]. Although the development of intensified chemotherapy has significantly enhanced the prognosis of ALL patients, the outcome of patients with relapsed or refractory ALL remains poor. For this reason, understanding the biology of the disease is crucial for the development of alternative approaches in order to achieve better therapeutic outcomes.

Cytokines are small soluble proteins secreted by specific cells of immune system and usually have a distinct effect on the interactions between cells [4]. Cytokines are an important part in immune responses induced by different infectious and non-infectious stimuli. In consonance with observations in other disease patterns, inflammatory microenvironment might prospectively promulgate leukaemia immune escape. Recent studies have demonstrated that abnormal expression of cytokines and/or activation of their receptors contribute to the onset and progression in several haematopoietic malignancies [5,6]. The expression of commonly studied cytokines such as vascular endothelial growth factor (VEGF), IL-6, and IL-10, were found to correlate with the outcomes of patients with chronic lymphocytic leukaemia (CLL), thus indicating the benefit of using cytokines as biomarkers for treatment efficacy monitoring [7]. According to a more recent study, the levels of inflammatory cytokines TNF-α and IL-6 in patients with ALL induces a T helper cell type 1 (Th1)- polarized response and suggests the presence of a pro-inflammatory profile in the cancer microenvironment in ALL patients at diagnosis [8].

We have previously reported elevated IL-33 levels in patients with acute myeloid leukaemia (AML) at diagnosis along with upregulated expression of its receptor, interleukin-1 receptor-like 1 (IL1RL1), as compared to the healthy controls. Further evidence indicates that IL-33 inhibits cell apoptosis by activating p38 mitogen-activated protein kinase (MAPK) pathway in both human AML cell line and AML patient samples, thereby supporting an important role of IL1RL1/IL-33 axis in maintaining AML development [9,10].

In the present study, we selectively analysed the expression of IL-33 and IL1RL1 in the serum and bone marrow (BM) samples of paediatric patients with ALL at diagnosis. We found that in comparison to healthy donors (HD), serum concentration of IL-33 is upregulated in ALL patients. More importantly, the expression of IL1RL1 is also higher in BM cells from ALL patient samples than those from healthy controls. By treating primary ALL samples with IL-33 *in vitro*, we found that IL-33 inhibited apoptosis and increased cell survival by activating the p38 MAPK and AKT. Meanwhile, the addition of p38 MAPK signalling inhibitor was found to abrogate the anti-apoptosis effect by IL-33 *via* inactivating the p38 MAPK/AKT pathway. Our findings suggest that IL-33/p38 MAPK/AKT pathway could represent a target for overcoming environment-mediated chemotherapy in ALL.

## 2. Materials and methods

### 2.1. Patients and patient sample collection

ALL patient samples (*n* = 15) were collected from diagnostic BM aspirations or serum after obtaining written informed consent. BM mononuclear cells (BMNCs) were isolated by Human Mononuclear Cells Separation Medium 1.077 (Dongfang Huahui) and cryopreserved until further use. BM and serum samples from healthy donors (HD) (*n* = 5) were collected in this study after obtaining informed consent. All experiments involving human samples were performed in accordance with the Declaration of Helsinki and approved from the Guangzhou Women and Children’s Medical Centre Ethics Committee. Information of ALL patients was provided in Supplemental Table S1.

### 2.2. Cell cultures

Primary ALL MNCs were cultivated in RPMI1640 (ATCC) supplemented with 20% foetal bovine serum (FBS), penicillin (50 IU/mL), and streptomycin sulphate (50 μg/mL), and 2 mM L-Glutamine. IL-33 (Sino Biological) was used at 100 ng/mL, and SB203580 used at 20 μM. All cells were grown at 37 °C and 5% CO_2_.

### 2.3. Enzyme-linked immunosorbent assay (ELISA)

To analyse serum IL-33 and secreted IL-33 levels in supernatants from cultured ALL patients or HD, human IL-33 ELISA Kit (4 A Biotech) was used in accordance with the manufacturer’s protocol. The samples and the standard samples were incubated with horseradish peroxidase (HRP)-conjugated antibodies. The substrate TMB was then added, which turned blue under the catalytic action of peroxidase. Absorbance was measured at 450 nm. The specific concentrations were calculated by the software program CurveExpert version 1.4.

### 2.4. Flow cytometry

After isolating MNCs from the BM of both ALL patients and HD, cells were stained with anti-IL1RL1 antibody. After that, around 1 × 10^6^ primary BM cells were incubated with antibody specifically recognising IL1RL1(BD Biosciences) for 1 h on ice and resuspended in phosphate-buffered saline (PBS) with 5% FBS. We used the FITC-conjugated Annexin V (Procell) and 4′,6-diamidino-2-phenylindole (DAPI) for the measurement of apoptosis and cellular viability. Data were analysed using CytoFlex S (Beckman Coulter) within 1 h. For cell cycle distribution, cells were fixed with ice-cold 70% ethanol for at least 30 min at 4 °C, before being treated with RNAse for 30 min at 37 °C. Subsequently, cells were subjected to propidium iodide (PI) staining and flow cytometry. At least 10^5^ events were collected for each measurement at medium flow (30 μL/min). FACS data was analysed by CytExpert 2.3 software (Beckman Coulter) and ModFit software (Verity Software). All experiments were repeated 4 times independently.

### 2.5. Western blot analysis

Cells were washed with ice-cold PBS and centrifuged at 400 × g for 5 min at 4 °C. The resulting pellet was lysed with RIPA buffer (KeyGEN) supplemented with 1x protease/phosphatase inhibitor (Solarbio). The homogenate was cleared by centrifugation at 4 °C for 10 min at 16,000 × g, and the supernatant containing the protein fraction recovered. Samples were subjected to electrophoresis in SDS-PAGE gels and transferred to PVDF membranes (Millipore). Membranes were blocked with 1% non-fat milk and incubated with primary antibodies overnight, followed by 1-hour incubation with HRP-conjugated anti-rabbit or anti-mouse secondary antibodies at room temperature and washed 3 times. The following primary antibodies were used: anti-p-p38 MAPK (Cat.#YP0338), anti-p38 (Cat.#YT3513) (Immunoway), anti-AKT (S473) (Cat.#A5030), anti-AKT (Cat.# 10176-2-AP) (Cell Signalling Technology), anti-CDK1 (Cat.# CY516-50), anti-GAPDH (Cat.# 12231 P) (Abways), and anti-cyclin B1(Santa Cruz Biotechnology). Antigen-specific binding of antibodies was detected with the ECL (enhanced chemiluminescence) Plus reagents (Beyotime). Protein bands were quantified ImageJ software (Wright Cell Imaging Facility) and then normalized to the GAPDH loading control. For full scans of Western blots see Supplemental Figures S1–S2.

### 2.6. Statistical analysis

Data are represented as mean values ± standard deviation. Statistical analysis was performed using the paired *t* test for two groups, and one-way ANOVA with Turkey post hoc test for multiple groups. Statistical significance is indicated by *p < .05, **p < .01. All analyses were performed in Prism 8.0.1 (GraphPad software Inc.).

## 3. Results

### 3.1. Il1rl1 cell surface expression and serum IL-33 levels are upregulated in patients with ALL

**Figure 1.**
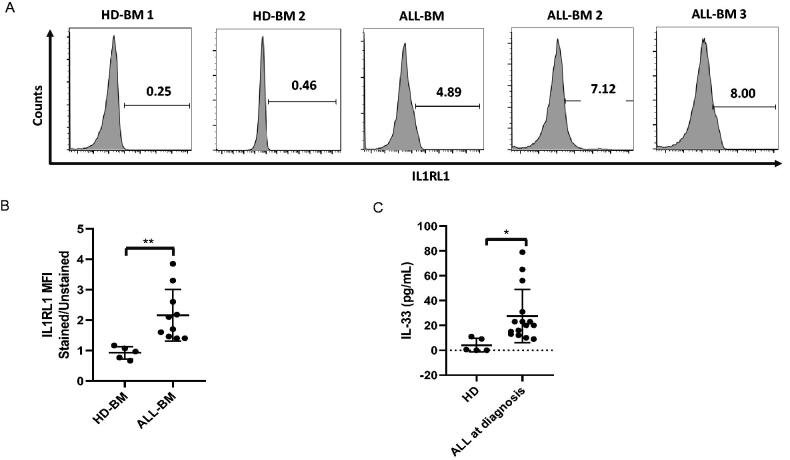
Primary ALL cells upregulate IL1RL1 cell-surface expression and serum IL-33 level. (A-B) IL1RL1 expression is increased on the cell surface of BM cells from ALL cohorts. BM cells from patients with ALL and HD were analysed for IL1RL1 expression by flow cytometry. (A) Histograms show two representative samples for each group of ALL patients (*n* = 10) or HD (*n* = 5). (B) IL1RL1 expression on primary ALL cells expressed as relative mean fluorescence intensity (MFI stained/MFI unstained). Each symbol represents one HD or patient with ALL. Paired *t* test. (C) ELISA assay was used to measure IL-33 levels in serum from ALL patients at diagnosis (*n* = 15) and HD (*n* = 5). Each symbol represents one HD or patient with ALL. Paired *t* test. *p < .05; **p < .01.

Although much effort has been put into exploring a cell-surface biomarker for leukaemia cells, no cell-surface marker has been shown to functionally differentiate leukaemia cells from normal cells. Since IL-33 is known to mediate its biologic effects by binding to IL1RL1, we first addressed the question as to whether IL1RL1 expression is upregulated in primary ALL samples. Flow cytometric analysis revealed a substantial IL1RL1 expression from ALL patients (*n* = 10) examined, while the vast majority of cells from heathy donors (*n* = 5) displayed this receptor at undetectable levels (Figure 1(A,B)). These data suggest that ALL cells may overexpress IL1RL1 to achieve a higher survival rate. To investigate and evaluate the potential clinical significance of serum IL-33 for ALL, we performed ELISA to measure the expression levels of serum in ALL patients (*n* = 15) and healthy controls (*n* = 5). According to our findings, the expression levels of serum IL-33 were significantly higher in ALL patients than the healthy control group (Figure 1(C)). Taken together, these results indicate that IL1RL1/IL-33 axis may represent a promising biomarker in ALL patients.

### 3.2. P38 MAPK pathway is activated in response to IL-33 and promotes survival of primary ALL samples

**Figure 2.**
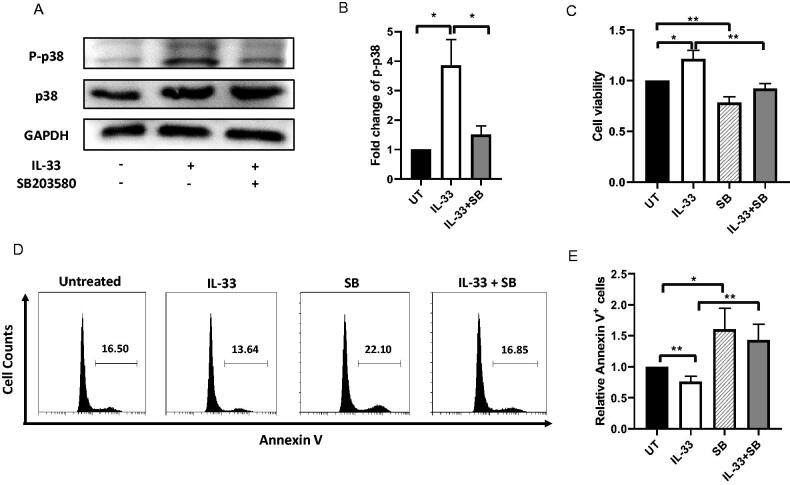
IL-33 promotes cell survival by activating p38 MAPK. (A) BM cells from ALL patients at initial diagnosis were incubated for 72 h with IL-33 (100 ng/mL) or SB203580 (SB, 20 uM) alone, or in combination. Cell lysates from each treatment group were prepared. P-p38, p38, or GAPDH protein expression was probed by western blot analysis. (B) The bar graph shows the relative quantification of p-p38 protein in all groups as compared to the untreated cells. One-way ANOVA with Tukey post hoc test. (C) Cellular viability was measured by DAPI staining. Bar graph shows the relative cell viability as compared to the untreated cells. One-way ANOVA with Tukey post hoc test. (D) Apoptosis was measured by Annexin V staining. Bar graph shows the relative Annexin V staining of leukaemia cells as compared to the untreated cells. One-way ANOVA with Tukey post hoc test. *n* = 4; *p < .05; **p < .01.

Previous studies have demonstrated that IL-33 stimulation can lead to the activation p38 MAPK cascade in different types of cells and modulate cellular activities, including cytokine production and apoptosis [11–13]. To examine whether p38 MAPK is one of the downstream signalling pathways stimulated by IL-33, we treated primary BM cells from ALL patients with IL-33, or in combination with SB in culture for 72 h. Western blot analysis showed that the level of p-p38 was markedly increased in IL-33-treatment group, as compared to the untreated cells. Meanwhile, the inhibition of p38 MAPK with SB led to abrogated phosphorylation of p38 (Figure 2(A,B)). To examine whether IL-33/IL1RL1 axis regulates viability or apoptosis of ALL cells *via* modulation of p38 MAPK activation, we stained cells with DAPI and analysed for viability by flow cytometry. We found that IL-33 significantly increased the cellular viability levels in comparison to untreated cells, while the addition of SB decreased the increased viability mediated by IL-33 (Figure 2(C)). In addition, we observed a significant decrease in annexin V expression in cells treated with IL-33 cells as compared to untreated group, and that SB caused a reversal of annexin V expression as compared to IL-33 alone, thus indicating that IL-33 led to a decrease in apoptosis, while inhibition of p38 MAPK pathway abrogated the anti-apoptotic effect mediated by IL-33 (Figure 2(D,E)). Importantly, cells treated with SB alone decreased viability while increasing apoptosis, as compared to the untreated samples. Therefore, our results suggest that activation of p38 MAPK pathway is involved in cell survival response mediated by IL-33 in primary ALL patient samples.

### 3.3. IL-33 promotes progression through G2/M phase via P38 MAPK

**Figure 3.**
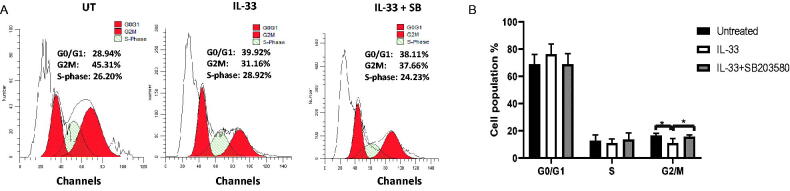
IL-33 regulates cell cycle progression *via* p38 MAPK signalling. BM cells from ALL patients were cultured with IL-33 (100 ng/mL), or combined with SB (20 uM) for 72 h and analysed for cell cycle status by flow cytometry. (A) Representative histograms show the cell cycle analysis of primary ALL cells from each treatment. (B) Bar graph shows the relative percentage of leukaemia cells in the indicated phase of the cell cycle. One-way ANOVA with Tukey post hoc test. *n* = 4; *p < .05.

Cell cycle arrest is one of the major causes of apoptosis. To examine whether IL-33 influences cell cycle progression *via* p38 MAPK pathway, we determined the distribution of cells in each cell cycle stage from the above treatment using flow cytometry. We found that IL-33 treatment effectively reduced the proportion of cells in the G2/M phase, while the combination of SB with IL-33 arrested cells at G2/M phase as compared with IL-33 treatment alone. It is also notable that IL-33 caused a trend towards an increase in G0/G1 phase, as compared to the untreated control, although this difference is not statistically significant. Moreover, the combination of IL-33 and SB decreased the proportion of cells in G0/G1 phase as compared to IL −33 treatment alone (Figure 3(A,B)). These results indicate that IL-33 regulates cell cycle progression *via* p38 MAPK pathway, while inhibiting the activation of p38 MAPK causes cell cycle arrest, which further leads to apoptosis in primary ALL patient samples.

### 3.4. IL-33 modulates cell cycle-related protein in primary ALL sample via the p38 MAPK/AKT

**Figure 4.**
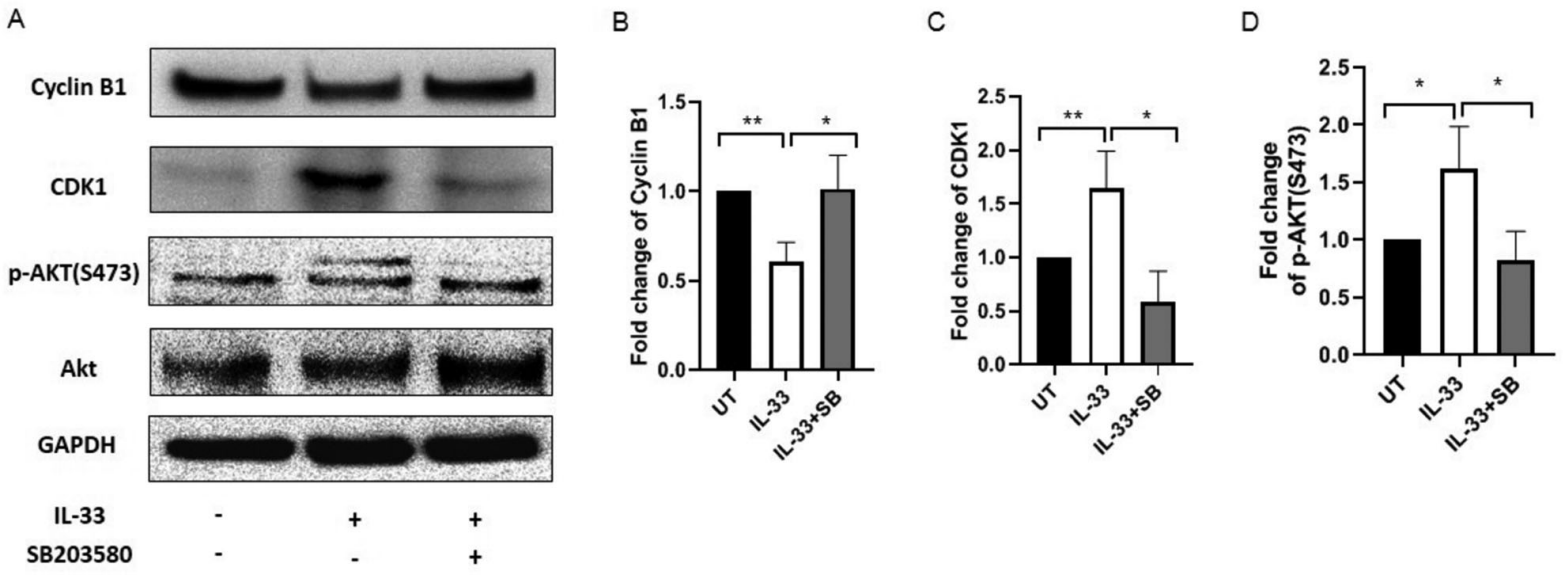
IL-33 modulates protein expression related to cell cycle *via* p38 MAPK/AKT. (A) BM cells from ALL patients were cultured with IL-33 (100 ng/mL), or combined with SB (20 uM) for 72 h. Cell lysates from each treatment group were prepared. CDK1, cyclin B1, AKT, pAKT (Ser473) or GAPDH protein expression was probed by western blot analysis. (B) The bar graphs show the quantification of pAKT, CDK1, and cyclin B1 protein in all groups. One-way ANOVA with Tukey post hoc test. *n* = 3; *p < .05; **p < .01.

Previous studies suggest that CDK1 and cyclin B1 are major checkpoint proteins of transition from the G2-phase to mitosis (G2/M), and a proper control of CDK1/cyclin B1 activity is absolutely essential for appropriate mitotic initiation and progression [14]. To assess the reason as to why IL-33 modulates cell cycle progression in primary ALL cells, the expression of CDK1, and cyclin B1 in the treated ALL cells (as elucidated above) was examined. IL-33 caused an increase in the level of CDK1 as compared to the untreated control, while the combination of SB with IL-33 reduced the level of CDK1 protein as compared to IL-33 alone. Interestingly, the expression of cyclin B1 was found to decrease under the treatment of IL-33 as compared to the untreated cells, while SB treatment significantly recovered the expression level of cyclin B1 caused by IL-33 (Figure 4(A–C)). Our results suggest that IL-33 may regulate cell cycle progression *via* modulating the expression of G2/M phase-regulating proteins.

AKT and MAPK pathways are known to be involved in various cellular responses, including cell proliferation, apoptosis, and metastasis [15,16]. To assess whether AKT pathway participates in IL-33-mediated cell cycle progression, we examined the expression levels of total AKT and phosphorylated AKT(Ser473) after treating the BM cells from ALL patients with IL-33, or in combination with SB203580 in culture for 72 h. We found that IL-33 significantly increased the phosphorylation of AKT compared with untreated cells, while the addition of SB abrogated the effect, indicating that AKT pathway is involved cell cycle status mediated by IL-33 *via* p38 MAPK (Figure 4(D)).

## 4. Discussion

ALL is the most common paediatric malignancy in childhood, arising from genetic aberrations that impair the normal differentiation of lymphoid cell lineage [17]. Although treatment has improved drastically over the past few decades, approximately 20–25% of paediatric patients with ALL still experience disease relapse [17,18]. Recent studies have shown that aberrant expression of cytokines and their receptors in the tumour microenvironment contributes to clonal expansion of transformed lymphoid precursor cells, thus promoting the progression of leukaemia [5,19–21]. IL-33 has been shown to participate in different biological activities in both nonhematological and hematological malignancies [22–25]. Using a knock-in mouse model of inversion (16) AML, we previously demonstrated that the fusion gene, *Cbfb-MYH11*, induces the expression of IL-33 receptor, IL1RL1, on leukaemia cells.

In the current study, we first showed that the IL1RL1 was constitutively expressed in BM cells from paediatric ALL patients at diagnosis, whereas BM cells from HD appeared to express IL1RL1 at very low or undetectable levels. Moreover, in accordance with the upregulated IL1RL1 on leukaemia cell surface, we observed a remarkably increased serum IL-33 level in primary ALL patient samples in comparison to HD. Therefore, it can be inferred that IL-33/IL1RL1 axis might potentially play an oncogenic role during the development of ALL. It is worthwhile to pointing out that serum IL-33 is a potentially useful tool as a significant prognostic factor for ALL. Further studies with a longer follow-up will be necessitated.

Cytokines are acknowledged to be common anti-apoptotic targets mainly through mediating cell signal transduction pathways [9,10,26]. A previous study demonstrated that IL-33 inhibited the apoptosis of AML cells by stimulating p38 MAPK pathway [9]. In support of the heightened expression of IL33 and its receptor in primary ALL samples, we found that the exogenous addition of IL-33 increased cell viability and decreased apoptotic level as compared with untreated cells. Importantly, the addition of p38 MAPK inhibitor, SB203580 (SB), inhibited the pro-survival effect mediated by IL-33. These results suggest that IL-33 enhances p38 MAPK pathway to support leukaemia development in ALL. Although it is not feasible to determine whether the anti-apoptotic role of IL-33/p38 MAPK pathway is applicable to all subtypes of ALL, our data provide fundamental data and the basis for further investigations on the biological mechanisms associated with IL-33/p38 MAPK signalling during ALL maintenance.

Additionally, it is also known that cytokines are important mediators for normal regulation of the cell cycle [27,28]. To decipher the mechanism of IL-33/p38 MAPK in regulating leukaemia cell survival, we assessed the cell cycle status in BM cells from children with first recurrence of ALL by flow cytometry. After treating the cells with IL-33 or in conjunction with SB for 72 h in culture, we found that IL-33 significantly decreased the cell proportion in G2/M phase, while SB resulted in a profound G2/M arrest. Moreover, by performing western blot, we found that the expression of two cell cycle-associated proteins, CDK1 and cyclin B1, was regulated by IL-33 *via* p38 MAPK with higher level of CDK1 and lower level of cyclin B1, as compared with the untreated cells. Importantly, we found that the phosphorylation of AKT was upregulated by IL-33, and that its expression was decreased with the treatment of SB. These findings indicate that IL-33/p38 MAPK/AKT axis regulates cell cycle by monitoring CDK1/cyclin B1 protein expression, thus implying an important role of IL-33 in controlling cell cycle and eventually causing a higher proliferation rate as well as a lower apoptotic rate in primary ALL samples. Furthermore, our observation was consistent to our previous study, in which IL-33 induced an increase of S-phase cells, thus promoting proliferation in both primary mouse leukaemia cells and patient AML samples [9,29]. Our findings are similar to the recent work in human osteosarcoma demonstrating that the induction of G2/M phase cell cycle arrest induces a decrease in G0/G1 phase as well as an increase in cell apoptosis [30]. Therefore, our findings open the door to the possibility of using pharmacological inhibitors of CDK1 in treating ALL. In addition, identifying specific inhibitors of IL-33/p38 MAPK/AKT pathway in conjunction with other therapies, such as chemotherapy, targeted therapies, and immunotherapy, may be further investigated so as to broaden the horizon of the biology of IL-33 in ALL.

**Figure 5.**
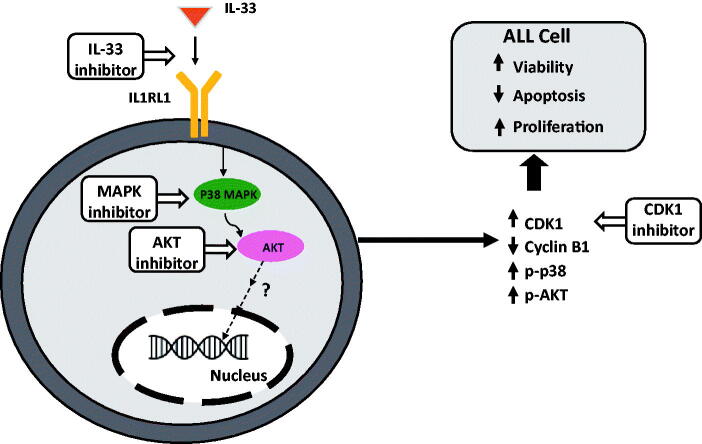
Proposed mechanism of IL33/IL1RL1 axis in mediating ALL cell survival by activating p38 MAPK.

In summary, we demonstrate that IL-33/IL1RL1 axis can lead to an abnormal activation of several downstream signalling pathways including p38 MAPK and AKT, which are capable of modulating cell cycle, inhibiting apoptosis and increasing cell viability. Inhibiting either p38 MAPK, AKT, or CDK1 can induce apoptosis of leukemic blasts, thereby contributing to cell death. Further studies will be required to delineate the molecular mechanism by which p38/AKT regulates downstream signalling and the expression of target genes to support ALL survival (Figure 5).

## Supplementary Material

Supplemental Material

## Data Availability

The data that support the findings of this study are available from the corresponding author, Qing Gong, upon reasonable request.

## References

[1] Tijchon E, Havinga J, van Leeuwen FN, et al. B-lineage transcription factors and cooperating gene lesions required for leukemia development. Leukemia. 2013;27(3):541–552. 23047478 10.1038/leu.2012.293

[2] Aldoss I, Stein AS. Advances in adult acute lymphoblastic leukemia therapy. Leuk Lymphoma. 2018;59(5):1033–1050. 28745565 10.1080/10428194.2017.1354372

[3] Siegel DA, Henley SJ, Li J, et al. Rates and trends of pediatric acute lymphoblastic Leukemia – United States, 2001–2014. MMWR Morb Mortal Wkly Rep. 2017;66(36):950–954. 28910269 10.15585/mmwr.mm6636a3 PMC5657918

[4] Kassab C, Kerrigan BP, Caruso H, et al. Chapter 15 – immunomodulatory methods. In: Lonser RR, Sarntinoranont M, Bankiewicz K, editors. Nervous system drug delivery. Cambridge: Academic Press; 2019. p. 297–334.

[5] Wu S, Korte A, Gessner R, et al. Levels of the soluble, 55-kilodalton isoform of tumor necrosis factor receptor in bone marrow are correlated with the clinical outcome of children with acute lymphoblastic leukemia in first recurrence. Cancer. 2003;98(3):625–631. 12879482 10.1002/cncr.11553

[6] Ivanoff J, Ivanoff A, Sundqvist KG. Defective chemokine production in T-leukemia cell lines and its possible functional role. Dev Immunol. 2000;7(2–4):67–75. 11097202 10.1155/2000/28085 PMC2276053

[7] Aguayo A, O’Brien S, Keating M, et al. Clinical relevance of intracellular vascular endothelial growth factor levels in B-cell chronic lymphocytic leukemia. Blood. 2000;96(2):768–770. 10887147

[8] Perez-Figueroa E, Sanchez-Cuaxospa M, Martinez-Soto KA, et al. Strong inflammatory response and Th1-polarization profile in children with acute lymphoblastic leukemia without apparent infection. Oncol Rep. 2016;35(5):2699–2706. 26985678 10.3892/or.2016.4657

[9] Wang Y, Su H, Yan M, et al. Interleukin-33 promotes cell survival via p38 MAPK-Mediated interleukin-6 gene expression and release in pediatric AML. Front Immunol. 2020;11:595053. 33324412 10.3389/fimmu.2020.595053 PMC7726021

[10] Wang Y, Luo H, Wei M, et al. IL-33/IL1RL1 axis regulates cell survival through the p38 MAPK pathway in acute myeloid leukemia. Leuk Res. 2020;96:106409. 32652328 10.1016/j.leukres.2020.106409

[11] Petrova T, Pesic J, Pardali K, et al. p38 MAPK signalling regulates cytokine production in IL-33 stimulated type 2 innate lymphoid cells. Sci Rep. 2020;10(1):3479. 32103032 10.1038/s41598-020-60089-0 PMC7044202

[12] Ochayon DE, Ali A, Alarcon PC, et al. IL-33 promotes type 1 cytokine expression via p38 MAPK in human NK cells. J Leukoc Biol. 2020;107(4):663–671. 32017227 10.1002/JLB.3A0120-379RR PMC7229703

[13] McCarthy PC, Phair IR, Greger C, et al. IL-33 regulates cytokine production and neutrophil recruitment via the p38 MAPK-activated kinases MK2/3. Immunol Cell Biol. 2019;97(1):54–71. 30171775 10.1111/imcb.12200 PMC6378613

[14] Yang Y, Xue K, Li Z, et al. c-Myc regulates the CDK1/cyclin B1 dependent-G2/M cell cycle progression by histone H4 acetylation in Raji cells. Int J Mol Med. 2018;41(6):3366–3378. 29512702 10.3892/ijmm.2018.3519 PMC5881754

[15] Lu Z, Xu S. ERK1/2 MAP kinases in cell survival and apoptosis. IUBMB Life. 2006;58(11):621–631. 17085381 10.1080/15216540600957438

[16] Mabuchi S, Ohmichi M, Kimura A, et al. Inhibition of phosphorylation of BAD and raf-1 by akt sensitizes human ovarian cancer cells to paclitaxel. J Biol Chem. 2002;6277(36):33490–33500. 12087097 10.1074/jbc.M204042200

[17] Pui CH. Recent advances in the biology and treatment of childhood acute lymphoblastic leukemia. Curr Opin Hematol. 1998;5(4):292–301. 9747636 10.1097/00062752-199807000-00009

[18] Szczepanek J, Styczyński J, Haus O, et al. Relapse of acute lymphoblastic leukemia in children in the context of microarray analyses. Arch Immunol Ther Exp. 2011;59(1):61–68. 10.1007/s00005-010-0110-1 21246408

[19] Wu S, Geßner R, von Stackelberg A, et al. Cytokine/cytokine receptor gene expression in childhood acute lymphoblastic leukemia: correlation of expression and clinical outcome at first disease recurrence. Cancer. 2005;103(5):1054–1063. 15651075 10.1002/cncr.20869

[20] Islam M, Mohamed EH, Esa E, et al. Circulating cytokines and small molecules follow distinct expression patterns in acute myeloid leukaemia. Br J Cancer. 2017;117(10):1551–1556. 28898234 10.1038/bjc.2017.316 PMC5680464

[21] Fayad L, Keating MJ, Reuben JM, et al. Interleukin-6 and interleukin-10 levels in chronic lymphocytic leukemia: correlation with phenotypic characteristics and outcome. Blood. 2001;97(1):256–263. 11133769 10.1182/blood.v97.1.256

[22] Levescot A, Flamant S, Basbous S, et al. BCR-ABL-induced deregulation of the IL-33/ST2 pathway in CD34+ progenitors from chronic myeloid leukemia patients. Cancer Res. 2014;74(10):2669–2676. 24675360 10.1158/0008-5472.CAN-13-2797

[23] Gao X, Wang X, Yang Q, et al. Tumoral expression of IL-33 inhibits tumor growth and modifies the tumor microenvironment through CD8+ T and NK cells. J Immunol. 2015;194(1):438–445. 25429071 10.4049/jimmunol.1401344 PMC4272901

[24] Lu B, Yang M, Wang Q. Interleukin-33 in tumorigenesis, tumor immune evasion, and cancer immunotherapy. J Mol Med. 2016;94(5):535–543. 26922618 10.1007/s00109-016-1397-0

[25] Casciaro M, Cardia R, Di Salvo E, et al. Interleukin-33 involvement in nonsmall cell lung carcinomas: an update. Biomolecules. 2019;9(5):203. 31130612 10.3390/biom9050203 PMC6572046

[26] Horita M, Andreu EJ, Benito A, et al. Blockade of the Bcr-Abl kinase activity induces apoptosis of chronic myelogenous leukemia cells by suppressing signal transducer and activator of transcription 5-dependent expression of bcl-xL. J Exp Med. 2000;191(6):977–984. 10727459 10.1084/jem.191.6.977 PMC2193112

[27] Quelle FW. Cytokine signaling to the cell cycle. Immunol Res. 2007;39(1–3):173–184. 17917064 10.1007/s12026-007-0080-5

[28] Van Etten RA. Aberrant cytokine signaling in leukemia. Oncogene. 2007;26(47):6738–6749. 17934482 10.1038/sj.onc.1210758 PMC4344827

[29] Wang Y, Richter L, Becker M, et al. IL1RL1 is dynamically expressed on Cbfb-MYH11+ leukemia stem cells and promotes cell survival. Sci Rep. 2019;9(1):1729. 30742053 10.1038/s41598-018-38408-3 PMC6370767

[30] Shangguan W-J, Li H, Zhang Y-H. Induction of G2/M phase cell cycle arrest and apoptosis by ginsenoside Rf in human osteosarcoma MG-63 cells through the mitochondrial pathway. Oncol Rep. 2014;31(1):305–313. 24173574 10.3892/or.2013.2815

