# Supplementary material for: P38 MAPK/AKT signalling is involved in IL-33-mediated anti-apoptosis in childhood acute lymphoblastic leukaemia blast cells
Source: Ann Med. 2021 Aug 26;53(1):1464–72. doi: 10.1080/07853890.2021.1970217 (PMC8405111; doi:10.1080/07853890.2021.1970217)
Supplement: Supplemental Material [file IANN_A_1970217_SM9690.pdf]

P38 MAPK/AKT signaling is involved in IL-33-mediated anti-apoptosis in childhood acute lymphoblastic leukemia blast cells

Yiqian Wang [1†], Hanyi Hou [2†], Zhongping Liang [3†], Xuexin Chen [1], Xindan Lian [4], Jie Yang [4], Zeyu Zhu [5], Huanmin Luo [6], Haibo Su [1*], and Qing Gong [1*]

ID	Age/Sex	ALL subtype	% Blast	% CD19$^+$	% CD3$^+$
1	2/M	B-ALL	75.5	93.4	-
2	10/M	T-ALL	95	-	81.6
3	7/M	T-ALL	76	-	87
4	4/F	B-ALL	85.5	99.1	-
5	4/M	B-ALL	76.5	99.1	-
6	1/M	B-ALL	70.5	99.1	-
7	1/M	B-ALL	70.5	99.5	-
8	3/F	B-ALL	87.5	99.2	-
9	3/M	B-ALL	70.5	98	-
10	6/M	B-ALL	52	97.7	-
11	4/M	B-ALL	85.5	99	-
12	2/F	B-ALL	78	99.1	-
13	2/M	B-ALL	91	91.7	-
14	6/M	B-ALL	70.5	99.2	-
15	7/F	B-ALL	75.5	99.9	-

Supplemental Table S1. Charateristics of Acute Lymphoblastic Leukemia (ALL) patients collected in this study.

Age is given in years at the time of initial diagnosis. F, female; M, male, -, undetectable.

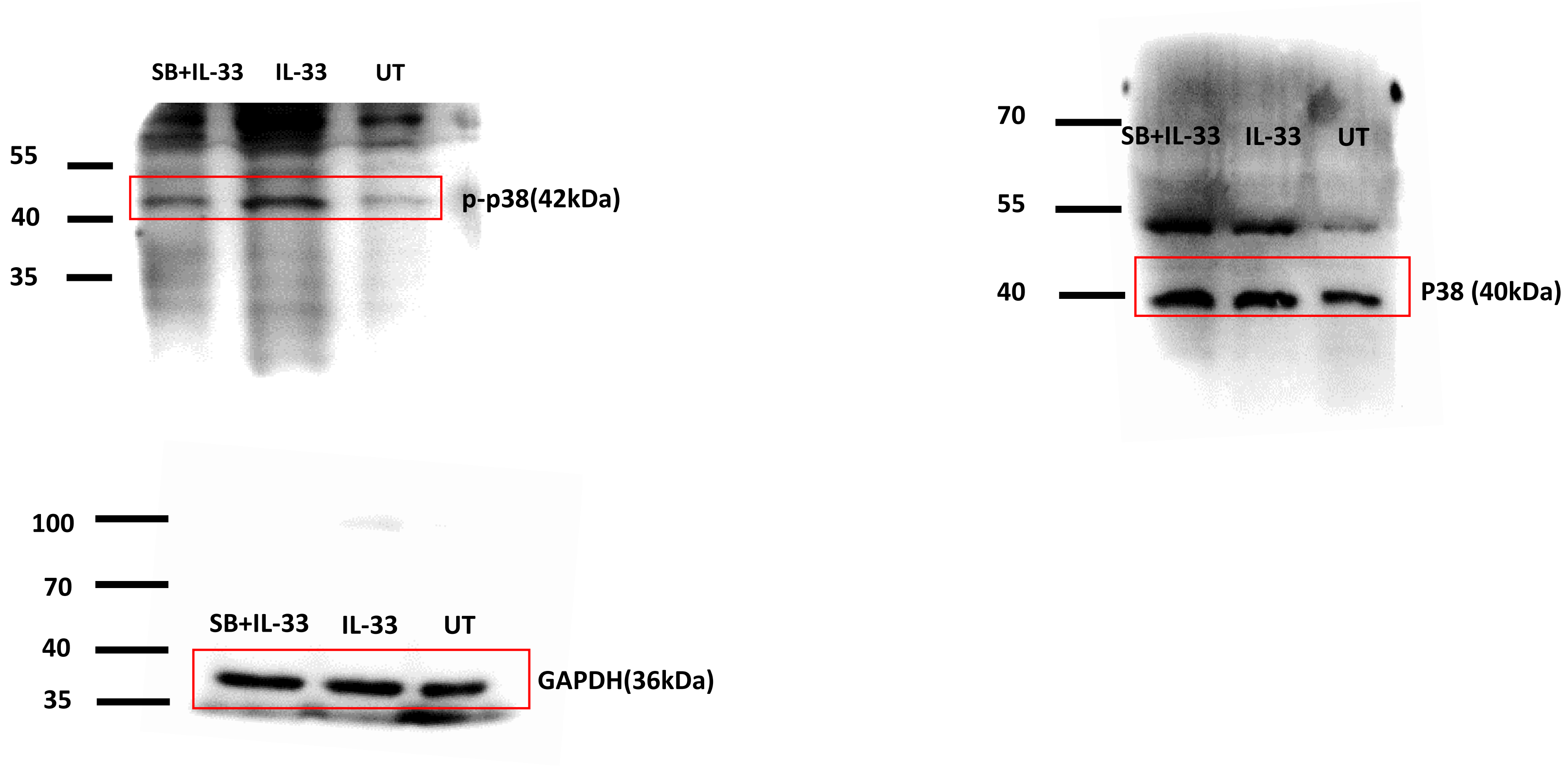

Supplemental Figure S1. Full size images of western blots shown in Figure 2A.

The cropped area (in red) corresponding to that shown in the main figure.

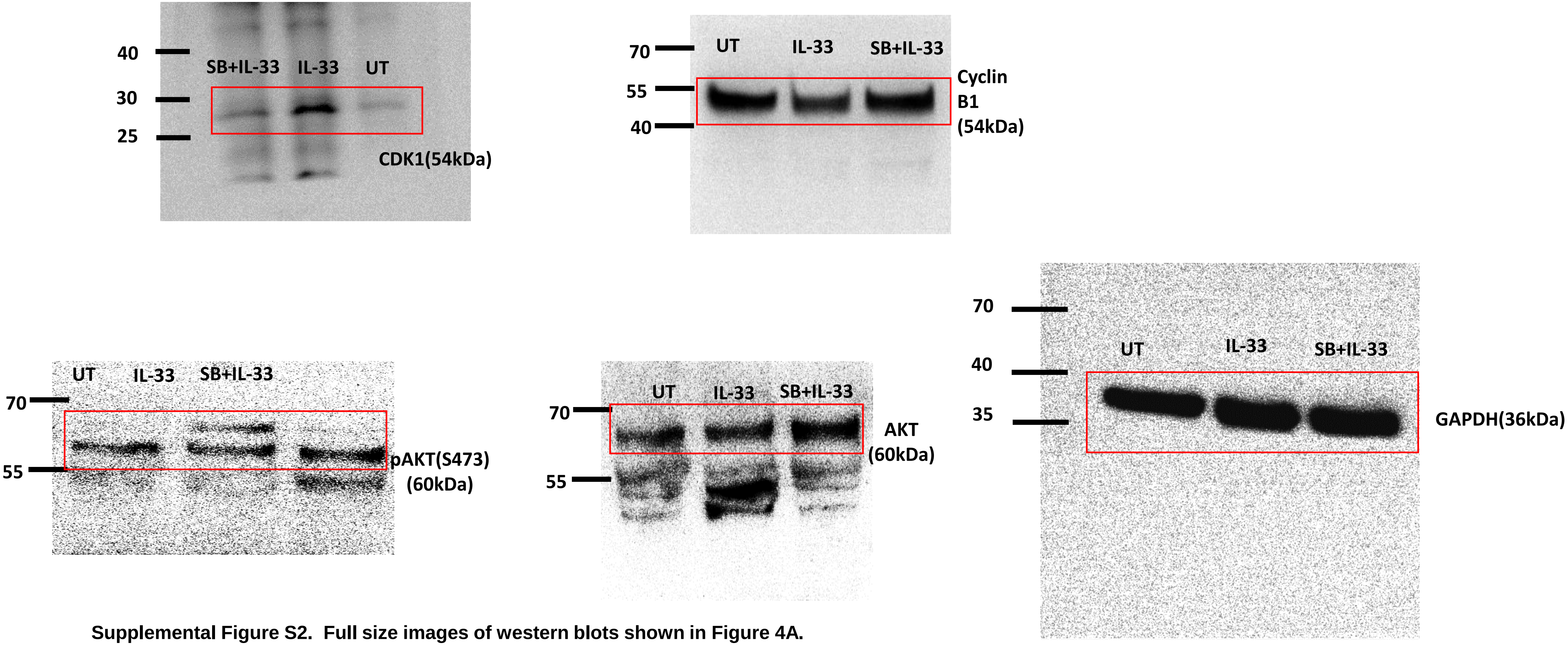

Supplemental Figure S2. Full size images of western blots shown in Figure 4A.

The cropped area (in red) corresponding to that shown in the main figure.
